# Implementation of a web-based system to measure, monitor, and promote school engagement strategies. A Chilean experience

**DOI:** 10.3389/fpsyg.2022.980902

**Published:** 2022-09-20

**Authors:** Mahia Saracostti, Ximena de Toro, Andrea Rossi, Laura Lara, María Belen Sotomayor

**Affiliations:** ^1^Cátedra UNESCO Bienestar de la Niñez y Juventud, Educación y Sociedad, Escuela de Trabajo Social, Universidad de Valparaíso, Valparaíso, Chile; ^2^Núcleo Científico Tecnológico en Ciencias Sociales y Humanidades, Universidad de la Frontera, Temuco, Chile; ^3^Departamento de Trabajo Social, Pontificia Universidad Católica de Chile, Santiago, Chile; ^4^Departamento de Psicología, Universidad Autónoma de Chile, Santiago, Chile; ^5^Departamento de Psicología Evolutiva y de la Educación, Universidad de Sevilla, Seville, Spain

**Keywords:** web-based system, platform evaluation, school engagement, evaluation and strategies, school web system implementation

## Abstract

This paper presents the implementation and usability of a technology-based web system and the available evidence on educational engagement’s predictive capacity to influence students’ educational trajectories in Chilean schools. The web-based system was developed through collaborative work between universities, the information technology team, school communities, and stakeholders (government institutions). It is an online system composed of six steps whose axis is centered on a decision-making space between teachers-students-parents (School Engagement Board) tasked with applying online and scientifically validated school engagement and contextual factors measurement instruments, checking specific report results for each actor involved in the system (teacher, School Engagement Board coordinator, school) as well as reviewing promotion strategies relevant to the school context and managing the implementation of strategies supported by the management datasheets that the model offers to schools. The objective of this paper is to present the usability of the system through a case study of the implementation in Chilean public schools. In order to discuss about what elements should be incorporated to adjust and improve the usability of the system and to guarantee its effective implementation, the paper describes those aspects that have favored and/or hindered the use of this educational technological platform in the Chilean case. The results show that there have been more difficulties related to management aspects than IT aspects, which indicates that these conditions are critical for implementation, even when system for evaluation, monitoring and strategies for the promotion of student engagement and contextual factors (SIESE) is designed for stand-alone use. Although there are aspects to be improved, such as extending its use to other browsers, improving the intervention guidelines and other systems functionalities, this web-based system has been considered by the educational communities as a simple, useful, and intuitive platform. The paper concludes on the importance of having this type of platform in Chile and other Latin American countries, for its contribution to school management -being helpful for day-to-day educational practice- due to the different technical facilitators.

## Introduction

### Monitoring systems for the promotion of successful educational trajectories

Quality education is a fundamental right and is part of the Sustainable Development Goals (SDGs). To achieve this goal, it is vital to promote school retention and prevent dropout. Monitoring systems are one of the recommended measures (Fredricks et al., 2019; Hofkens and Rozek, 2019; de Toro et al., 2021). They are referred to as educational trajectory protection systems or early warning and intervention systems. Regardless of their denomination, prevention and promotion only occur if detection is accompanied by a set of measures. UNESCO (2021) highlights how these systems can become effective tools for reducing dropout, and the importance of linking them with other educational management systems.

Monitoring systems are an essential element to evaluate program and processes, to identify strengths or weaknesses in schools, to contribute to decision-making and carry out educational management processes at different levels, for example at the school, local or government level (Komar et al., 2019). The Chilean Ministry of Education in its Framework for Good School Management and Leadership establishes a specific dimension for the monitoring process, indicating that management teams should monitor the comprehensive implementation of the curriculum and learning achievements in all areas of student training for the improvement of teaching and pedagogical management processes (Leiva-Guerrero et al., 2022). Likewise, Local Educational Services, also by law, must have follow-up, information and monitoring systems that address the evaluation of the processes and results of educational institutions, as part of the continuous improvement of the quality of education.

UNESCO (2021) notes that there are two monitoring systems. The first is a model based on expert knowledge or indicators that identify the most at-risk students. A second model is based on data analysis using machine learning techniques that process large amounts of data to identify trends in the aggregate behavior of indicators by schools or geographic areas. This second model is used when student-level data is not available. While in the models based on expert knowledge there are successful experiences using a few indicators, the other models require a large amount of information, including quality historical data, to produce good results. Moreover, as the latter models use a large amount of data, the information obtained may not be easily understandable. For this reason, the first models are more recommendable.

### Indicators with scientific evidence

A fundamental component of monitoring systems is to have scientifically validated indicators that provide schools with clues about what decisions to make to improve the educational trajectories of students. School engagement (SE) is one of the key indicators since abandonment is not usually a sudden act, but rather the final stage of a cumulative process of a loss of commitment to studies (Miranda-Zapata et al., 2018, 2021; Fredricks et al., 2019).

School engagement is considered a primary concept to promote school retention and protect educational trajectories. SE can be defined as the active participation of the students in the educational process, while they are motivated and consider their learning as meaningful. SE is a multidimensional construct with an affective, a behavioral and a cognitive dimension. Affective dimension refers to the level of emotional response of the child toward the educational institution and their learning process, characterized by a feeling of involvement with the school and a consideration of it as a place worth being part of. Behavioral dimension includes student interactions and responses within the classroom, school, and extracurricular settings. Finally, cognitive dimension is the conscious investment of energy to build complex learning that goes beyond the minimum requirements (Shernoff, 2013; Fredricks et al., 2016, 2019; Christenson et al., 2018; Lara et al., 2018; Miranda-Zapata et al., 2018; Saracostti et al., 2019, 2021).

This concept has become even more relevant in times of pandemic and post-pandemic, as remote classes were installed as an alternative modality in the face of the uncertainty of the behavior of COVID-19, with attendance losing strength as an indicator of educational processes (Zhao and Watterston, 2021).

On the one hand, SE is a variable highly influenced by Contextual Factors (CF), mainly by (i) family support; (ii) peer support and (iii) teacher support (). Family factor refers to the fact that students perceive family support in the learning process, in case of problems, with homework, in motivational terms, together with conversations about what happens at school. Pers support has been defined as the perception that students have about interpersonal relationships between classmates, that is, the concern, trust and support that is given between peers. Finally, teacher support refers to the fact that students perceive receiving support and motivation from teachers to learn or in case of any inconvenience (Saracostti et al., 2019; Navarro et al., 2021).

On the other hand, the literature, together with international and national organizations, agree that having systems capable of evaluating, monitoring and promoting the school engagement contribute to prevent school dropout and promote positive trajectories in a much timelier manner than those initiatives that focus exclusively on the classic risk factors such as absenteeism or falling behind in school. The relevance of focusing on school engagement lies in the fact that it is a highly moldable variable and on which schools can intervene, unlike more structural factors such as poverty. Schools and local entities can be benefited from an integrated and articulated system for promoting SE and FC, since it could identify common needs among students and courses that require additional support, while governments Local authorities can focus resources on those schools or areas that need to reinforce their SE, together with evaluating the effectiveness of the available interventions. It is also suggested that these systems be based on robust technical components that do not increase the teaching load (Bruce et al., 2011; de Toro et al., 2021).

### Robust technical components

Collecting data on indicators associated with the risk of dropping out is not enough to prevent school dropout. It is necessary a methodology for this purpose (UNESCO, 2021), to have robust technical components and to be connected to a network of interventions (de Toro et al., 2021). Regarding the use of data for the improvement of educational processes, Parra and Matus (2018) recommend having digital registration systems that systematize information and deliver indicators over time, so that strengths and alerts can be quickly and timely identified; and on the other hand, to follow up in the different educational periods. The web-based school system software automates school operations and helps to reduce management burden.

Based on an incremental scientific-technological development, the Web-Based System to Measure, Monitor, and Promote School Engagement Strategies (SIESE), easily and quickly accessible at https://compromisoescolar.com/ allows measuring, monitoring and intervening on the basis of automated reporting in favor of School Engagement (SE) and the Contextual Factors (CF) which influence it of students in educational transition from 5th grade (or 5th grade of primary school, 10−11 years old) to 12th grade (secondary school or 2nd year of high school, 17−18 years old) (de Toro et al., 2021).

System for evaluation, monitoring and strategies for the promotion of student engagement and contextual factors has been perfected and adjusted to the reality of diverse educational community contexts to integrate on-site the requirements for its proper use. For this reason, throughout the incremental scientific-technological development, based on different R + D + i projects, which resulted in the SIESE, platform operation was evaluated and adjusted according to the suggestions and observations for improvement of the participating educational communities of the country (de Toro et al., 2021). In addition, SIESE contains scientific validations of (i) measurement tools of SE and FC (Lara et al., 2018, 2021, Navarro et al., 2021; Sotomayor et al., 2021), (ii) predictive model of the Effect of School Engagement on Attendance to Classes and School Performance that is at SIESE base of operation (Miranda-Zapata et al., 2018), and (iii) strategies proposed (Leyton et al., 2021).

System for evaluation, monitoring and strategies for the promotion of student engagement and contextual factors was developed using SCRUM methodology (de Toro et al., 2021), which is a lightweight software development methodology that relies on incremental development and focuses on delivering several iterations of a product. It was developed through collaborative work between the university team (researchers), information technology team, school communities, and stakeholders (government institutions).

Technical details of the software and hardware that supports the different components of the technological platform, which should be considered at the time of installation and transfer, are detailed as follows:

• *User Layer*: The client application for accessing the measurement instruments, intervention strategy record cards, and downloading reports are executed through a Web browser installed on the accessing computer, preferably Chrome or Firefox. Mobile accessibility stands out in this layer so that participants can access the surveys and reports using mobile devices such as tablets or smartphones to facilitate the application and distributed follow-up.
• *Applications Layer*: Consisting of the server where the modules for authentication, measurement instruments, reports and longitudinal monitoring, and strategies and record datasheets for the actions implemented in the respective classes and schools are installed. The measurement instruments in an online format were developed based on LimeSurvey technology that uses the Yii (PHP) development framework as a base. In the case of the results report visualization module, these were developed using HTML, JavaScript, CSS, and PHP development environments.
• *Data*: Consisting of the database in which the information of each participant in the measurement process is stored, as well as the profiles associated with the management of the platform and the educational resources available (audios, videos, documents, etc.). The database implemented the MySQL engine, running on a centralized, high-performance server (24/7) and available in the cloud (Internet) under Linux operating system and Apache Web server.

### Background: Use of system for evaluation, monitoring and strategies for the promotion of student engagement and contextual factors through its 6 steps

System for evaluation, monitoring and strategies for the promotion of student engagement and contextual factors, which is based on the Early Warning System developed by the National High School Center of the American Institute for Research (AIR, 2020), proposes the sequential execution of 6 steps: composed of 6 steps: (i) Step 1: Formation of the SE Board; (ii) Step 2: Measurement of SE; Step 3: Review and analysis of the information; Step 4: Selection and implementation of strategies; Step 5: Monitoring of students and strategies; Step 6: Evaluation and adjustment of the implementation of the System (de Toro et al., 2021).

Each step is briefly described below, as well as some guidelines for their execution (Tables 1–7):

**TABLE 1:** Implementation of step 1: System for evaluation, monitoring and strategies for the promotion of student engagement and contextual factors (SE) board formation.

Description step 1: SE board formation	Guiding questions
Participative constitution of a decision-making space within the school to carry out the implementation. The SE Board can be by class, educational level or school and it is desirable that it be made up of students, teachers, psychosocial professional teams, families and management teams. The participation of students and families is fundamental and representatives of other projects that work in the school to promote positive educational trajectories can also be involved.	1. Who will be part of the Board? 2. Does the Board include the participation of students and families? 3. What other representatives that support the students within the school could join? 4. Who will be the coordinator responsible for convening the meetings and completing the registration forms included in the system? 5. Does the Board have sufficient authority to implement changes, and what will it take to get it to do so? 6. What resources or strategies does the school have to promote SE?

**Specific resources**	**Expected results**

-Training (Complementary Material module with educational resources, hosted in the same web system). -Activity Calendar (annual schedule of 10 meetings).	-1st Meeting of the School Engagement Board to choose a School Engagement Board coordinator and plan the annual meetings. -Training of the participants of the Board through the Complementary Material module.

Source: own elaboration based on https://compromisoescolar.com/.

**TABLE 2:** Step 2: SE measurement.

Description step 2: CF and SE measurement	Guiding questions
It is expected that in this step a strategy will be organized to communicate the objective of the SIESE to the rest of the school community. Subsequently, the idea is that students can answer the SE and CF measurement instruments. The system allows to apply the measurement instruments once a year, in a classroom context (computer room) or remotely (zoom or similar). The SE Board coordinator has to activate the code so that students can respond. It is suggested that responses can be collected within 1 week.	1. How do we sensitize the entire educational community on the importance of SE? 2. How will we promote the application of the SE and CF instrument among the students, considering whether we are in a face-to-face, hybrid or non-face-to-face context? 3. Who will be responsible for the promotion? 4. Who will explain to the students how to answer the SE and CF instrument in this platform? 5. Where will the application take place? 6. What strategies will be implemented to ensure that those who have not completed the SE and CF instrument will be able to do so?

**Specific resources**	**Expected results**

-User manual of the platform. -SE (29 items) and CF (18 items) measurement instruments, created and validated with good levels of fit for Chile and later for other Ibero-American countries (Lara et al., 2021) with 5-point Likert-type response scales (1 = Never or almost never, 2 = Sometimes; 3 = Often, 4 = Many times and 5 = Always or almost always).	- 2nd Meeting of the Board to coordinate a strategy to promote SE among students, along with the meaning of measuring SE. - Measurement of School Engagement. - Review of the percentage of instruments answered. - 3rd Meeting of the SE Board to coordinate additional strategy and assign responsibilities to ensure that students who have not completed the instrument are able to do so. - Review of the percentage of final responses.

Source: own elaboration based on https://compromisoescolar.com/.

**TABLE 3:** Step 3: Review and analysis of the information.

Description step 3: Information review and analysis	Guiding questions
This step involves downloading and reviewing the results reports by grade that this platform produces in an automated manner based on algorithms (Saracostti et al., 2021), which are protected by intellectual property confidentiality. There are reports according to the different SIESE access profiles The individual reports are reserved for the exclusive use of teachers and/or psychosocial team and not for the work of the SE Board. Then, it is expected that all the members of the SE Board actively participate in the analysis and interpretation of the data by grade, complementing the quantitative information provided by this platform with background information provided by families, students and teachers, which will allow a better understanding of the results. This diagnosis is of vital importance as an initial baseline for decision making regarding the selection of strategies to be applied.	1. Who will download the reports from the platform for the SE Board? 2. What other indicators can be analyzed to complement the information (e.g., performance, behavior, attendance)? 3. What information does the platform provide? 4. What needs emerge from the analysis of the results? 5. What school or classroom practices can be linked to the results shown on the SE platform?

**Specific resources**	**Expected results**

-Platform User Manual. -Reports of SE and CF measurement results at student, grade and school level.	-Coordinator downloads the results reports. -4th SE Board meeting to analyze reports and identify causes and needs associated with the results.

Source: own elaboration based on https://compromisoescolar.com/.

**TABLE 4:** Step 4: Selection and implementation of the SE promotion strategies.

Description step 4: SE promotion strategy selection and implementation	Guiding questions
In this step, the SE Board has to identify the most relevant intervention strategies for the grade and the school, as well as for the individual students. This analysis will be recorded in the first datasheet called “Action Plan.” To do so, you can enter the SIESE strategy search engine where you will find a set of datasheets with guidelines for the promotion of SE and CF. These strategies are based on international evidence, reviewed by teachers and school coexistence teams in Chile and adapted to the national context The Board will have to select those strategies that are most relevant to promote the different types of SE (affective, cognitive or behavioral) and CF (family, teachers or peers). Other interventions already identified or implemented in the school or in other schools can also be enhanced. It should be addressed how the strategies will be implemented and which other actors should be incorporated in their application (e.g., other teachers and/or professionals).	1. What existing strategies in our school are relevant to foster SE and what could be enhanced to address the needs reported by this platform? 2. What strategies described in this platform could be implemented in our school? 3. What steps do we need to follow to manage the implementation of the strategy? 4. What additional actors to the SE Board need to be involved in the implementation of the strategies? 5. How to engage their participation?

**Specific resources**	**Expected results**

-Strategy search engine -SIESE datasheets: Grade action plan (Figure 4). -SIESE datasheets: Individual datasheet.	-Review the intervention strategies available on the platform (23) and select the one or ones most relevant to the school context (Table 5). -Fourth meeting of the SE Board to complete the Action Plan Worksheet (Figure 4). - Planning strategy management.

Source: own elaboration based on https://compromisoescolar.com/.

**TABLE 5:** Summary of the strategies proposed in the SIESE, according to SE dimension and Contextual Factors.

SE dimension	Universal	Targeted	Individual
Affective	- Parental counseling - Connecting learning with real life - Regular contact with fathers, mothers and guardians - Joint goal setting - Peer-to-peer collaborative learning strategies - Encouraging family involvement - Improving school climate - Promoting restorative practices - Promoting empathy - Acknowledging student voice - Rethinking extracurricular activities	- Strengthening social-emotional skills to reduce bullying - Strengthening student participation in classes - Identifying learning needs in a collaborative context - Pedagogical tutoring	- Regular contact with parents and guardians - Identifying learning needs in a collaborative context
Behavioral	- Parental counseling - Promoting restorative practices - Promoting empathy		- Student-led family interview - Problem-solving technique
Cognitive	- Parental counseling - Connecting to the future of work - Connecting learning to real life - Promoting growth mindset - Informed feedback	- Learning on a project basis - Identifying learning needs in a collaborative context - Pedagogical tutoring	- Identifying learning needs in a collaborative context

**CF dimension**	**Universal**	**Targeted**	**Individual**

Family	∙ Parental counseling ∙ Regular contact with parents and guardians ∙ Encouraging family involvement ∙ Promoting restorative practices		∙ Regular contact with parents and guardians ∙ Student-led family interview ∙ Problem-solving techniques
Teachers	∙ Connecting learning to real life ∙ Emotional connection ∙ Strengthening student participation in class ∙ Improving school climate ∙ Promoting a growth mindset ∙ Acknowledging student voice	∙ Emotional connection ∙ Strengthening student investment in learning ∙ Identifying learning needs in a collaborative context ∙ Pedagogical tutoring	∙ Identifying learning needs in a collaborative context
Peers	∙ Emotional connection ∙ Joint goal setting ∙ Peer-to-peer collaborative learning strategies ∙ Improving school climate ∙ Promoting restorative practices ∙ Rethinking extracurricular activities	∙ Emotional Connection ∙ Strengthening social-emotional skills to reduce bullying	∙ Problem-solving techniques

Source: https://compromisoescolar.com/estrategias. Reproduced with permission.

**TABLE 6:** Step 5: Selection and implementation of strategies for the promotion of SE.

Description step 5: Selection and implementation of strategies for the promotion of SE	Guiding questions
In this step the focus is for the SE Board to make a recurrent follow-up of the strategies implemented, reviewing the need for adjustments. This can be done by complementing their analysis with the opinions of other actors implementing the strategy and by reviewing other indicators, such as performance and attendance. Assess the need to make adjustments and record what changes are observed in the classroom, including changes in performance, attendance or behavior.	(1) How are the strategies being implemented? (2) What adjustments are needed? (3) How should adjustments be made? (4) What should each member of the SE Board do to ensure that the strategy or strategies are being implemented?

**Specific resources**	**Expected results**

-Strategy Search Engine. -SIESE Datasheets: Follow-up and Monitoring Datasheets (Figure 5).	-5th, 6th, 7th, 8th, and 9th Meeting of the SE Board to review the progress in the implementation of the selected strategy, review other action alternatives and/or make the necessary adjustments to the implemented actions. -Complete the Follow-up and Monitoring Sheets (Figure 5).

Source: own elaboration based on https://compromisoescolar.com/.

**TABLE 7:** Step 6: SIESE implementation assessment and adjustment.

Description Step 6: SIESE Implementation Evaluation and Adjustment	Guiding questions
At the end of the school year, an analysis of the process (learning, difficulties and obstacles) is suggested, as final feedback for the following year’s implementation cycle. It is expected that the SE Board will be able to make informed decisions to continue strengthening SE in their students.	(1) What were the main results of the strategies implemented? (2) How was the SIESE implementation process? (3) What were the main facilitators and/or obstacles? (4) What improvements can be made in the implementation of the SIESE for the next year?

**Specific resources**	**Expected results**

-SIESE Datasheets: Closing Datasheet (Figure 6)	-10th Meeting of the SE Board to analyze the process. -Complete Closing Sheet (Figure 6)

Source: own elaboration.

Those who are participants in the SE Board, as well as any other member of the educational community who so desires, can be trained through the same platform in the Complementary Material module by means of seven short videos, publications on conceptual and methodological aspects, technical documents, seminar and/or webinar records, frequently asked questions, among other resources. The SIESE also has a User’s Manual, a guiding document to optimize its use.

Once the SE Board is constituted, the schools can lead the second step, which is related to the process of applying the measurement instruments (Table 2).

Once the students have answered the SE and CF questionnaires, the SE Board can analyze the data recorded in the platform and establish a diagnosis (Table 3).

System for evaluation, monitoring and strategies for the promotion of student engagement and contextual factors provides annual (annual measurement) and follow-up (or longitudinal) reports for cases where more than one measurement has been made, at the school, grade and student level. The following is a description of how the data are presented in each of these reports, considering the graphs to support their interpretation.

#### Annual reports: At student level

**FIGURE 1:**
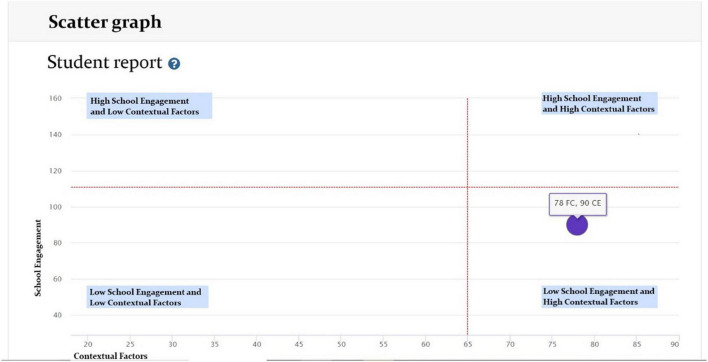
Example of results by system for evaluation, monitoring and strategies for the promotion of student engagement and contextual factors (SE) and contextual factors (CF) dimensions of the Student Web Report (scatter graph). Source: https://compromisoescolar.com/. Reproduced with permission.

**FIGURE 2:**
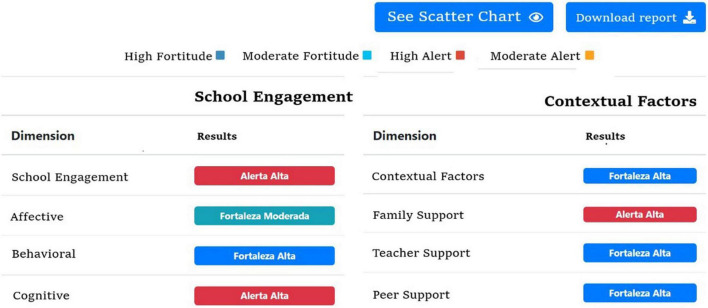
Sample of SE and CF Alerts and Strengths results from the Student Web Report. Source: https://compromisoescolar.com/. Reproduced with permission.

The analysis of results per student can be based on two readings: through the scatter graph (Figure 1) considering four possible profiles (high SE and low CF; high SE and high CF; low SE and low CF; low SE and high CF) and through the SE and CF graphs (Figure 2), which allow identifying signs of strengths and alerts, without showing scores in order to avoid stigmatizing boys, girls and adolescents [BGA] (Saracostti et al., 2021).

Once teachers and/or education professionals and the SE Board Coordinator review both types of graphs, it is suggested to take note of the most relevant aspects for later discussion at the SE Board. The interpretation of student reports is of vital importance for targeted and/or individual interventions.

#### Annual reports: At grade level

In addition to the scatter graphs that place each grade on axes of intersection of high and low levels of SE and CF, the reports by grade also contemplate pie charts that represent the percentage of students in each dimension of SE and CF at four levels of SE development (emerging, developing, satisfactory, highly developed) and four levels of CF (low, medium, high and very high) (Saracostti et al., 2021).

The report by grade also allows visualizing the concentration of students per item of each SE dimension (e.g., “When I am studying, I write down new words, doubts or important ideas,” “I feel that I am important to the school,” or “I behave in class”) and CF (e.g., “I talk to my family about what I do at school,” “My classmates support me and care about me,” or “Teachers care about me not only as a student but also as a person”), according to each response level (Never or Almost Never, Sometimes, Often, Many Times, Always or Almost Always).

#### Annual reports: At school level

Each educational institution can have scatter graphs of four profiles similar to Figure 1, which allow locating the levels of SE and CF, considering all the grades that participated in the measurement (Saracostti et al., 2021).

#### Monitoring or longitudinal reports: For multiple schools (for educational administrators), by school, grade level and student

**FIGURE 3:**
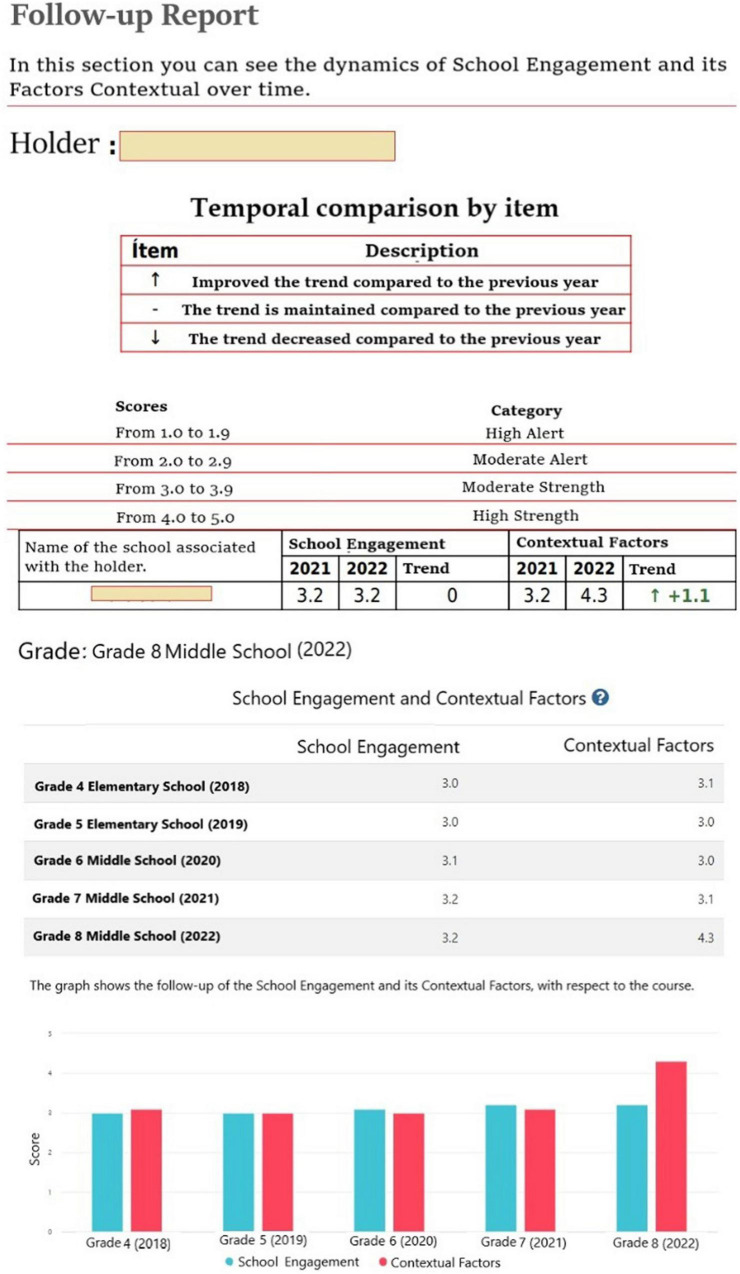
Examples of follow -up reports: “Administrator” Profile [A]. “Educational Institution” [B] Profile follow-up report. Source: https://compromisoescolar.com/. Reproduced with permission.

Longitudinal reports make it possible to analyze over time, establish trends and follow up on the grouped or individual behavior of students in order to identify changes in advance and intervene in a timely manner (Figure 3). Considering also that the SE and CF measurement instruments were created and validated for students from 5th to 12th grade, it would be possible to monitor and follow up during an important period of the students’ school career (Saracostti et al., 2021).

At the student and grade level, it is possible to access results reports similar to those shown in Figure 3.

Once the data has been analyzed through the different accesses to results that SIESE allows the SE Board must socialize this information to develop a collaborative decision-making process in order to promote SE and CFs of its students.

To support the educational communities in this process, the platform offers guides to the actors involved in the selection and implementation of strategies that are relevant to their school context (Table 4).

Once the search for strategies relevant to the results reflected in the reports provided by the SIESE has been carried out, it is possible to access explanatory sheets of the SE and CF promotion strategies, in web and downloadable versions, in order to select the most appropriate ones for the school context and student needs (Leyton et al., 2021). In addition, if you prefer to know all the strategies at once, it is possible to access a downloadable compendium (Table 5).

Each strategy indicates the sub-dimension of the SE and/or the CF it seeks to enhance. Each strategy sheet also indicates whether or not it is applicable in a non-face-to-face context, and whether its implementation is individualized (with one student), targeted (with a group of students) or universal (with the entire class or school) (Table 6).

**FIGURE 4:**
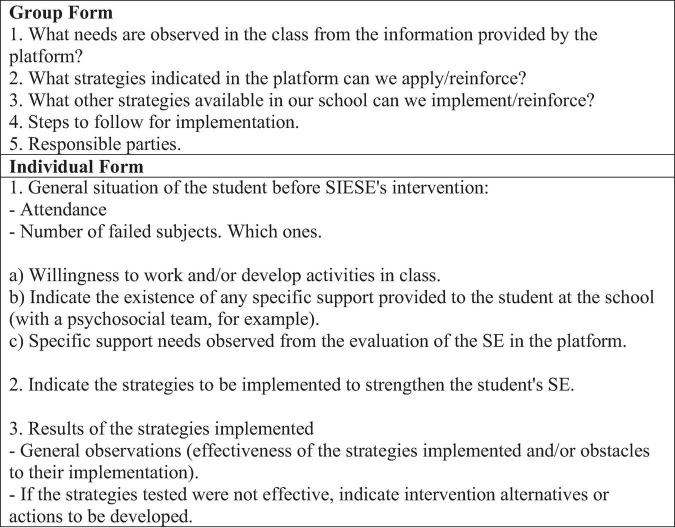
Items to be completed in the action plan worksheet. Source: own elaboration based on https://compromisoescolar.com/.

Once the strategies have been selected, the SE Board can fill in the “Action Plan” sheet (Figure 4) with the interventions identified as relevant to be implemented in their school context.

**FIGURE 5:**
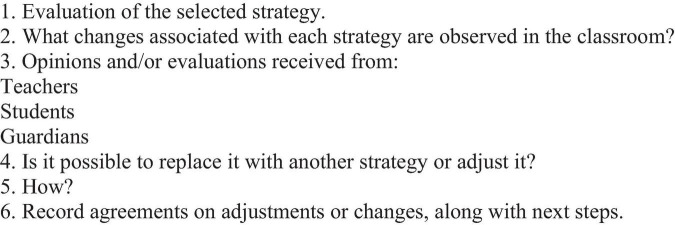
Items to be completed in follow-up and monitoring datasheets. Source: own elaboration based on https://compromisoescolar.com/.

Although SIESE is based on the comprehensive perspective of education as a universal right, it also allows, in a complementary manner, the registration of targeted interventions in cases that merit it. In relation to this last level, the use of the Individual SIESE Form supports decision making for specific student situations that require a more personalized intervention. In the same way, confidentiality and ethical management of the data entered in the Individual SIESE Form must be ensured (Figure 5).

**FIGURE 6:**
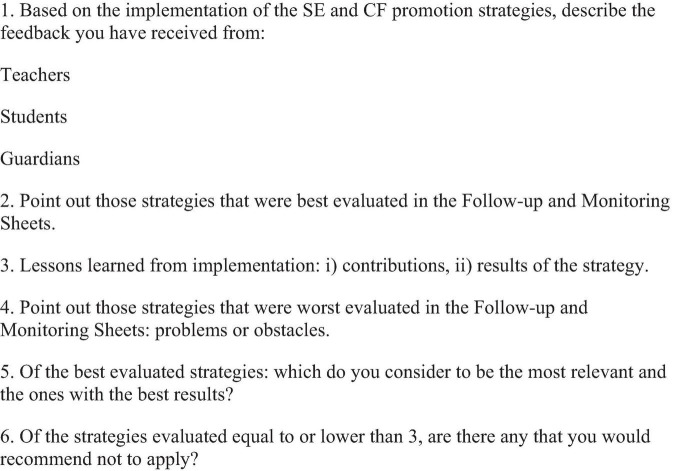
Items to be completed in closing datasheets. Source: own elaboration based on https://compromisoescolar.com/.

This Individual SIESE Form records that the intervention actions should be oriented to the promotion of CF. Complementarily, it is possible to evaluate joint actions associated with other management systems present within each school. The last step of the implementation of the SIESE system includes the final evaluation and adjustment, as seen in Table 7 and Figure 6

## Materials and methods

### Design

This paper shows the results of a qualitative and descriptive multiple case study. A case study allows to explore different parts of a phenomenon, and the way in which events, actors and structures converge in the same space (Yin, 2018). Each case is understood as a school in which the SIESE was applied throughout the school year. Being a case study, in-depth contact with singular realities was privileged over a large number of individuals as a source of information.

### Sample

The study considered six public schools with a high level of school vulnerability (about 90%) in two regions of Chile (V and VI Regions) belonging to the country’s central macrozone. The V Region of Valparaíso is the third most populated region in Chile, mainly urban and 19% of its population is in multidimensional poverty. The VI Region of Libertador Bernardo O‘Higgins represents 5.2% of the national population (sixth most populated in Chile), with a 25.6% rural population. The poverty rate (7.9%) is higher than the national rate (6.3), although multidimensional poverty is lower (18.5%) than the Chilean average (20.7%).

In Chile, public school students come from families with lower incomes and lower socio-cultural capital, which represents the segregation of the Chilean educational system. A high level of school vulnerability is equivalent to <85%. School Vulnerability Index (IVE, in Spanish) calculated annually by the National School Aid and Scholarship Board (JUNAEB, 2005), which ranges from 0% to100%, where the higher the percentage, the higher the vulnerability index. This percentage indicates the proportion of students defined as priority students (JUNAEB, 2005; Brunner, 2006). This index is measured according to poverty conditions and risk of school failure, including variables such as family socioeconomic context, access to the health system, housing quality, and parents’ educational level, among others. For school recruitment, this index is important as it is directly related to exclusion and dropout factors.

This is a convenience sample, (Please review the participant sample in Table 8), as it considers those schools that meet the vulnerability criteria and with which the research team has worked in previous studies.

**TABLE 8:** Participant sample.

	Students who responded to the survey	Total school enrollment	Professional Who responded to the interview	Data Sheet of the Observation Process	IVE	Region
School 1	96 (3 classes)	1200	1	1	90%	V Region (Urban area)
School 2	48 (2 classes)	511	1	1	97%	V Region (Urban area)
School 3	40 (2 classes)	235	1	1	91%	V Region (Urban area)
School 4	50 (2 classes)	314	1	1	91%	VI Region (Urban area)
School 5	54 (2 classes)	336	1	1	96%	VI Region (Urban-rural area)
School 6	39 (2 classes)	344	1	1	97%	VI Region (Urban-rural area)
Total students	327 (13 classes)	2940	6	6		

Source: own elaboration.

### Procedure

A socialization strategy, in which SIESE was presented to a large number of schools and school administrators was carried out to recruit schools that would be part of the study. Six schools were selected that voluntarily decided to participate in the SIESE application.

The schools implemented SIESE during the 2021 academic year (in the context of the pandemic quarantine) at the 7th (12−13 years) and 8th (13−14 years) grade levels. These educational levels were chosen because in Chile they correspond to the educational transition between primary and secondary education. Likewise, in the pre-pandemic context, the highest level of dropout was mainly at the next level (1st grade or first year of secondary school), so identifying the variables that influence this phenomenon was relevant if it was focused on the levels prior to this educational transition.

As a first step, schools were contacted to sign a commitment to participate. Each child responding to the survey was asked to sign an informed assent, while parents were asked for their consent. The pilot schools supported the formation of a School Engagement Board charged with implementing the six steps of the SIESE. During the first semester all coordination and applications were done virtually, while in the second semester they were done in person. The accompaniment was done through telephone, zoom and/or face-to-face meetings mainly with the coordinators of school engagement boards.

The project was approved by the ethics committee of the Universidad Autónoma de Chile (CEC 10−20).

### Techniques used

Data collection was qualitative in nature through the technique of participant observation of a member of the research team who accompanied the implementation of the SIESE during the school year. Participant observation is understood as a technique that involves interaction between the researcher and the informants (Taylor and Bogdan, 1984), during which data were collected in a systematic and non-intrusive manner.

During the observation process, data sheets were used (1 for each school), which were to be completed by the field researchers for each step of the SIESE implementation process and allowed collecting information on aspects that facilitated and/or hindered the proper development of each implementation phase, related to: (i) the use of the platform, (ii) emerging doubts, (iii) autonomy in the implementation of SIESE based on the information provided, (iv) emerging computer problems; as well as suggestions for improvement. This was complemented with the development of semi-structured interviews to 6 school professionals (1 of each school) who participated actively in the implementation of SIESE, through the School Engagement Board.

### Data analysis

The information collected from the data sheets of each implementation process, interviews and the experience of the research team was analyzed using the technique of thematic analysis. Following Braun and Clarke (2006), thematic analysis is understood as a method to identify, analyze and report patterns from the data, exploring common patterns, congruities and incongruities among participants. Table 9 describes the dimensions that were taken as reference at the time of the analysis:

**TABLE 9:** Analysis dimensions.

Dimension	Sub-dimension
Application of SIESE	Step-by-step implementation
	Facilitators (IT/management)
	Obstacles (IT/management)
	Adjustment needs (IT/management)
Evaluation of SIESE	Benefits or positive aspects Difficulties in implementation

Source: own elaboration.

## Results

The following is a description of the results obtained from the follow-up of the six schools that implemented SIESE along four lines: facilitators, obstacles, opportunities for improvement and suggestions for its implementation. The above, considering both the IT and management aspects of SIESE.

### Facilitators for system for evaluation, monitoring and strategies for the promotion of student engagement and contextual factors implementation

#### Resources to support the implementation of system for evaluation, monitoring and strategies for the promotion of student engagement and contextual factors

To facilitate the implementation of SIESE, a manual was developed to promote capacity building and autonomous management. In addition, participants had access to the training module which, based on suggestions from the educational institutions, was changed from an online course to a module of interactive videos of approximately 3 mins each. According to field researchers *“reports the development of audiovisual material was chosen due to the difficulties associated with the availability of sufficient time for reading*” (data sheet, school 2).

#### Internal organization of schools

With respect to the formation of the School Engagement Board and the planning of the sequential process proposed by SIESE, participant observations point out that: “*implementation was easier in those schools with a high degree of autonomy and self-initiative, recognizing this cultural aspect as a facilitator at the organizational level*” (data sheet, school 6). Considering field notes, “*previous existence of School Councils facilitated the creation of the SE Board*” (data sheet, school 4), as well as “*the motivation of teaching teams to incorporate new strategies in their curricular designs*” (data sheet, school 1).

#### Leadership committed to system for evaluation, monitoring and strategies for the promotion of student engagement and contextual factors

The coordination of teams within a school is a key aspect to consider for the development of SIESE steps. In this context, although it is not an element of SIESE itself nor is it linked to its usability, participant observations indicate that: “*the leadership characteristics of management teams, reflected in strategies for convening teaching teams and ability to socialize SIESE, motivating its integration and development, is considered a facilitator to ensure the implementation of the steps*” (data sheet, school 3). This was also relevant to facilitate student participation and understanding during the application of the measurement instrument. In addition, according to interviews, faced with emergent situations during the measurement process, in cases where “*there was more than one teacher to support the process, it was possible to better meet the different requirements*” (interview, school 6) such as verbal communication with students, the projection of the sequence of steps to follow on screen, as well as the attention to doubts and support via chat, the copy of codes and the review of the platform to corroborate the completed surveys.

#### Mass uploading of students

Schools received the students’ access keys to enter the platform (tokens), which were generally distributed through institutional e-mail. In this regard, it is worth noting the possibility of the platform performing mass uploads of students to cover a larger population of students and achieve nationwide coverage. This is a key aspect referenced in interviews. The users of the teaching and management teams must ensure the correct entry of the access codes to avoid problems during the application of the instruments and explain the process well: “*At the beginning it was a bit confusing because it was applied to all the classes together and the children were confused with the codes*” (interview, school 1).

#### Instruments easy to use by the students

Field workers reports evidence the application of the assessment instruments was further facilitated by the students’ familiarity with the technological platforms and because these were developed into a responsive version. Thus, they were able to access the instrument from a computer, tablet or mobile phone. “*There were no major difficulties for students to answer the instrument, which was understandable and quick to apply*” (data sheet, school 3). The results of the surveys also make sense to them: “*The data coincide with reality, as they match with other measurements carried out (for example, in relation to school coexistence problems*” (interview, school 6), except for the family support factor, whose interpretation generated certain misunderstandings as it was the factor with the fewest items in the survey.

#### Simple, useful, and intuitive platform

From participant observations, school teams highlighted the ease of use of SIESE in its navigation, being highly valued the platform, the forms and the information provided in the reports to support the work of teachers in school retention: *“Excellent platform, very practical and intuitive” (data sheet, school 6). “The platform is different from all the previous ones; it has functions that allow to record changes and actions. It provides guidance with good strategies. It has a methodology that allows to guide the action and making a good assessment”* (interview, school 5). “*I liked SIESE, it was very well used by the psychosocial team that was able to articulate the different interventions that are carried out by that team*” (interview, school 2).

They emphasize that reports are useful as they contribute to school management: “*The results show aspects not previously observed in the school; for example, that students feel supported at school. The survey has made it possible to objectify data on certain perceptions of the school’s teaching and management teams*” (interview, school 4). The fact that it is intuitive facilitates the autonomous use of SIESE, although its autonomous use without an advisory research team is undoubtedly a challenge and a line of research for future projects.

### Obstacles in the use of system for evaluation, monitoring and strategies for the promotion of student engagement and contextual factors

Obstacles were more related to the management of SIESE, since technological difficulties visualized throughout the process were improved.

#### Formation of school engagement boards

Their consolidation was difficult, as schools and teachers had a high demand for additional tasks due to the pandemic. There were also difficulties in meeting regularly according to the proposed schedule. This is something registered among field reports. In this regard, school professionals alluded to “*difficulties in meeting as the implementation of SIESE did not anticipate the professional hours assigned within the school*” (data sheet, school 5). In addition, there were complexities due to the high level of teacher absenteeism, associated with medical leaves, which implied for the schools a series of adaptations such as merging classes due to lack of teachers and being in permanent solution of contingency problems. Finally, in some cases, there were difficulties in holding meetings with all the members of the Boards, having to hold separate meetings in some cases with teachers, parents, and students to analyze the results and select strategies, since it was difficult to coordinate with everyone at the same time.

#### Participation of students and parents

Although SIESE model stresses the importance of children and parents being part of the decision-making team, their participation was more of a consultative nature: “*the students participated, but we had to ask them for their opinion*” (interview, School 5). “*There was a good intention of the Boards actors to guarantee student participation and to listen to their opinions, but most of the interaction was between adults*” (data sheet, school 4). This shows certain difficulties in developing spaces for intergenerational interaction and decision-making in a virtual context, and the importance of integrating strategies that allow the active participation of students and parents, since their participation was highly valued when they were effectively included: “*children were able to participate, see their interests and collaborate; this motivated them. It also made it possible to recognize changes in them, after some strategies*” (interview, school 3).

#### Dedicated time

The high work overload of teachers eroded the continuity of certain periodic activities proposed by the SIESE, such as reviewing materials, recording and analyzing information in a more systematic way for making decisions regarding the implementation and follow-up of strategies (fields reports). In this sense, the school’s experience points out “*the need to resort to the preparation of materials and activities for the implementation of strategies, which requires management teams*” (data sheet, school 6). This complexity was less preponderant in those cases where there was prior knowledge and integration of some strategies in schools.

#### Connectivity

According to participant observations, “*connectivity is a variable to consider when planning and developing the SIESE implementation in schools*” (data sheet, school 3), taking into account this obstacle of an external nature when children answered the instrument from their homes due to the pandemic, where they did not have a good internet connection. This point may also be an obstacle for rural schools.

### Aspects to improve

#### Extending its use to other browsers

The platform can only be used from the Chrome browser, which was an inconvenience for some users since this requirement was not explicit in the application instructions. On the other hand, field reports observed that, of the total sample, “*a very small number of students were unable to answer the survey because they could not pass the CAPTCHA validation*” (data sheet, school 1) associated with entering the password.

#### Improving the intervention guidelines

Datasheets and interviews stated that the strategies proposed by SIESE are useful since they are relevant to the school reality: “*Greater interaction and participation among students is observed, developing analysis sessions with the classroom teacher and the psychologist with topics related to routines and study habits*” (interview, school 6).

“*Parents express the importance of participating in activities that are for their academic benefit and integral development, and that are also necessary for everyone, especially at this age where adolescents sometimes become discouraged, losing interest in studies*” (interview, school 4).

However, there is a perceived need for more guidelines for the results analysis phase and for the selection and implementation of strategies. From participant observations, it is suggested that “*there should be a guide or a model of the strategies in SIESE to facilitate their implementation without wasting time in creating activitie*s” (data sheet, school 2). Suggestions for immediate intervention could also be incorporated into the reports.

#### Interpretation of the reports

In order to bring the results closer to students and parents, the interviewers suggested expanding the type of reports available to include versions that can be easily analyzed by students and parents in classrooms or at parent-teacher meetings. It is also suggested to specify in greater detail “*how to interpret the statistics presented for teaching teams that were not familiar with the graphs in the reports*” (data sheet, school 3), and to specify how the report can guide decision-making in schools.

#### Biannual application

The platform only allows an annual application per course, but as a suggestion, the possibility of applying at the beginning and at the end of the school year to see how school engagement changes from the implementation of SIESE is raised: “*We could start with the evaluation in March (beginning of the academic period in Chile), to measure how the year is going*” (interview, school 2).

#### System functionalities

Other suggestions made by the pilot (by interviews and field reports) are: to add a school search engine to facilitate the search by school administrators with a large number of schools under their responsibility (data sheet, school 2); that a teacher can be associated to two schools given that there are those who work in different educational institutions (data sheet, school 3); to allow longitudinal monitoring of the student regardless of transferring to another school. It is also suggested that the system allow “*to upload supporting evidence documents*” (interview, school 4).

### Suggestions for implementation

#### Teams with hours assigned to system for evaluation, monitoring and strategies for the promotion of student engagement and contextual factors implementation

It is a fundamental requirement that the School Engagement Board coordinators have hours assigned to SIESE implementation within the schools, which is facilitated if SIESE is part of the school’s management policy: *“It is advisable to assign professional hours to the project*” (interview, school 1). To ensure the execution of the activities proposed by SIESE for its optimal implementation, participants’ observations suggest scheduling the School Engagement Board meetings for the entire year, organizing in advance the subsequent events after the first meeting is held (data sheets, school 1 and 5).

#### Transfer to different actors

In addition to the school user, there must be a general administrator, which can be a local entity that groups together different schools. In this case, a model of transfer of the platform to the Ministry of Education was chosen, but it is possible to think of a transfer model composed of public or private non-profit actors to facilitate its mass use and support to schools. On the other hand, given the importance of updating the platforms as technology advances or in the face of changes in public policies, a transfer model was developed that allows the Ministry to make the necessary modifications so that it can be updated as technology and the use of the software advances. To facilitate the transfer, a technical guidelines document was also developed that includes examples of how to use SIESE according to the different access profiles (users) of SIESE. This document also specifies the pedagogical roles of all the actors of the educational community involved, as well as guidelines and/or suggestions for the interpretation and use of the data, in order to favor capacity building and autonomy for the implementation of SIESE in schools.

#### Continuous training

Although the platform allows self-training, users valued instances of support and/or face-to-face training to optimize its use. This is a key aspect collected in participant observations (data sheets, school 3, 4, and 6).

#### Supervised application of the instruments

Regarding the application of the instruments, according to interviews and field reports, some students needed more support and clarification regarding the correct entry of the codes (without spaces, with or without periods between numbers and initial letter) (data sheets, school 2 and 5). Therefore, it is recommended that the application of the instruments be done with an adult in charge to resolve doubts and during class hours for mass application. In this line, although the SIESE is designed to be used both in face-to-face and distance classrooms, participant observations note that “*in non-face-to-face instances, the application of the instruments may take more time than initially estimated and/or agreed upon*” (data sheet, school 4). Based on this, the importance of involving parents and student representatives to motivate their children and classmates, respectively, is emphasized.

#### Follow-up of the action plan

A risk observed in the experience of *Step 5* has been the non-implementation of the strategies planned to be applied in the “Action Plan.” To this end, we insist on the recommendation to ensure the implementation of the follow-up meetings suggested in the SIESE to review the planning and adjust, if necessary, according to the real needs and possibilities of the schools, making effective use of the monitoring sheets hosted on the platform. It was also suggested that the Action Plan Worksheet should allow “*Adding evidence, uploading supporting documents, attendance data, audiovisual files*” (interview, school 4).

#### Continuity over time

Given that the changes in SE and CF after the implementation of the strategies cannot necessarily be visualized in the short term, it is important that the implementation of the SIESE covers more than one semester, ideally the whole academic year, to ensure all instances of follow-up and to glimpse certain effects and/or impacts after the intervention. In this sense, it is observed, and interviews reaffirm that to the extent that the strategy or strategies tested (at the grade level) are complemented with changes at the school level and individualized strategies, it is likely that more changes will be perceived between one implementation and another (data sheets, school 3 and 6).

#### Reinforce roles for system for evaluation, monitoring and strategies for the promotion of student engagement and contextual factors implementation

Other suggestions resulting from participant observations are in line with what has already been pointed out in the SIESE, such as encouraging the participation of students and parents, together with “*reinforcing the role of school administrators and management teams for the socialization of the SIESE, motivating the rest of the members of the educational community and providing resources, mainly in terms of the time required, for example, by the Coordinator of the SE Board, and for the management of the interventions*” (data sheet, school 2). With this, favoring and/or ensuring the distribution of tasks so that responsibilities are shared among the members of the Board and not only charged by the Coordinator. This is key when completing the “Action Plan” worksheet.

## Discussion

System for evaluation, monitoring and strategies for the promotion of student engagement and contextual factors implementation of educational software faces challenges that require consideration to ensure effectiveness in its use. Based on the experiences described in this article, four challenges related to effective SIESE implementation in schools stand out. Each of these challenges is briefly discussed below.

### Requirements for technological installation and articulation with other educational management software

The school engagement platform corresponds to a Web solution consisting of six stages, allowing the application of surveys, deployment of reports, intervention record cards, and storage of pedagogical resources.

Considering this background, a central requirement for system installation is access to connectivity and use of the Internet. Regarding this, we highlight that in the case of Chile, the progression in Internet access has been dramatic: from 16% of the population in 2000 to 82% in 2018. Considering household access, it has gone from 32% in 2009 to 87% in 2017. The access gap between urban and rural areas has also decreased, from a difference of 27% in 2009 to 12% in Brújula Investigación y Estrategia (2017). Despite these advances, these studies also find that there are still differences in access by socioeconomic level, geographic areas, and age.

The school engagement web system has been developed and validated for autonomous management by schools; however, some improvements are still required. On the one hand, it is necessary to consider that to make modifications or improvements to the web system, in terms of the production environment, support from a computer expert is required. Some of the system’s challenges arise from the fact that some of an engineering team’s current actions could be conducted by education professionals. For example, to facilitate editing of measurement instruments or reporting format to make it easier and unnecessary to involve a technical engineering team.

Another central challenge for the installation is the articulation and communication between the school commitment software and the hardware technological infrastructure where the software works, as well as the articulation with other educational management platforms available in the public institutions and the schools. In this last aspect, for some users, data integration with other databases available in the schools, such as attendance or school performance databases, became relevant.

### Usability of the platform and support for school management in decision making

As stated by Hu (2022), a good application of educational information systems can contribute to the efficiency of educational management, to which Luan (2014) adds by guaranteeing the secure recording of information. In this context, SIESE stands out for its ability to provide school management with the possibility of articulating in the same information management system, measurement (quickly and massively), monitoring, and intervention at diverse levels: universally (establishment), targeted (course) and personalized (student). In this context, the challenge of any technological system is presented, and it is related to the ability to be used simultaneously by an important number of entities or people (multiple users). Luan (2014) defines this as *concurrency.*

In this sense, SIESE’s usability − highlighted by the experiences in the 6 Chilean public schools that participated in its implementation − favors school management since it can be used massively and quickly, we must not lose sight of aspects such as those indicated by the IDB (2021) about the Educational Information and Management Systems [SIGED] for Latin America and the Caribbean, for example, having a long-term strategic vision and responding to the needs of the educational system, ensuring interoperability between platforms and applications, and the availability of both economic and human resources for their development and implementation.

Now, most of the systems of this type available in the region aim at providing information for the central level. Not many platforms are helpful for day-to-day school practice, like monitoring students and/or teachers (IDB, 2021). In this case, the SIESE represents an added value since it provides relevant information for school decision-making (Schildkamp, 2019; Soncin and Cannistrà, 2022), either at the circumstantial or longitudinal level.

Likewise, the internal generation of data and the collaborative analysis on which SIESE is based constitute a benefit concerning other current measurements that commonly respond to isolated indicators or data, generated from external evaluations often perceived by Chilean educational communities as belonging to a system focused on accountability (accountability or accountability for results) (UNESCO, 2017; Riquelme et al., 2018). On the contrary, the SIESE approach highlights the importance of a non-standardized, non-prescriptive, and formative logic that encourages collective practices in data use through reflective rather than instrumental cycles. In this sense, the functionality of SIESE could approach a technological approach more pedagogical than administrative, or of internal utility from the data generated (UNESCO, 2017) and thus, make more sense to the educational community members and favor its appropriation.

However, agreeing with Selfridge (2018), another challenge lies in using and interpreting the data provided by educational software such as SIESE. It calls for the importance of having training processes that ensure the installation of capacities of the teams in schools in this area, for example, to understand quantitative data, know how to interpret diverse types of graphics, link them together, and how they can be used in favor of pedagogical processes. As Schildkamp (2019) states, data becomes helpful when it is transformed into information that can be applied through actions that benefit the context.

By Parra and Matus (2018), Soncin and Cannistrà (2022), reflective use of data in educational entities is highly desirable not only to favor decision making but also to encourage continuous improvement of pedagogical practices in favor of students’ learning and their educational trajectories. This is what the SIESE proposes conceptually and methodologically at its base.

### Technical-pedagogical support: Socialization and training

Although the platform incorporates training modules of diverse types to support the implementation process autonomously, from the case studies, the support provided is in the form of technical-pedagogical accompaniment (OEI, 2018) to conduct the different steps of SIESE is generally and positively recognized (OEI, 2018).

From the results, the need for this support given the complexities of resources, for example, the time and excessive workload for teachers, can also be confirmed. The results also make it clear that there have been more difficulties related to management aspects than IT, indicating that these conditions are critical for implementation even when SIESE is designed for stand-alone use.

In addition, given the stated need to incorporate more support resources or guidance on the use of the SIESE sheets, Schildkamp (2019) also emphasizes the relevance of socialization processes, whether external and/or internal, from the management teams and training that can provide an induction to encourage the efficient use of the platform.

### Educational community participation is a transversal axis of implementation

The platform proposes an interdisciplinary team approach (eco systemic perspective) with the involvement of various stakeholders from the educational community (Saracostti et al., 2020); however, in the case studies, the community was not always fully represented.

In addition, the analysis and decision-making process involved adults, mainly members of the schools. In particularly vulnerable contexts, parents’ lack of experience in being part of school decision-making or sitting next to principals and teachers is a complexity endemic to the Chilean education system. In general, decisions are delegated to the school. Added to this is the adult-centeredness of our society, a framework that the SIESE encourages to model.

Therefore, the composition of the CE Board constitutes a challenge regarding the configuration of a new cultural paradigm in the educational community. This requires the generation of mechanisms to promote the effective participation of students and parents in the CE Board. Given this context, it is imperative to raise awareness about these instances as non-bureaucratic but democratic spaces with clear goals and clear roles within the Board. An emphasis should be placed on the role that children and adolescents play as protagonists and decision-makers in their processes (Collins et al., 2021). Along the lines analyzed by Petrone et al. (2020), in this type of instance, there is an opportunity to overcome adult centrism (UNICEF, 2013).

Given that SIESE presents proper usability from the schools’ experience, for it to be effectively implemented and contribute -as proposed- to educational management, it is necessary to consolidate participation and collaborative decision-making between teachers-students-families. In this sense, management aspects re-emerge as key conditioning factors and challenges adjacent to the technological aspects.

## Final conclusion

One of the biggest educational challenges in Latin America is to develop systems that protect educational trajectories (IADB Blog, 2020). In this context, having integrated systems for measuring, monitoring, and promoting school engagement strategies, such as SIESE (Saracostti et al., 2019; de Toro et al., 2021), becomes a relevant and timely tool for the region’s challenges, which have been reinforced by the impact of the COVID’s socio-health crisis.

The most recent published literature (Fredricks et al., 2019; Hofkens and Rozek, 2019; AIR, 2020) agrees on the appropriateness of generating systemic proposals that promote measurement, monitoring, and intervention strategies in a not only targeted but uniquely universal manner, through promotional and preventive strategies for groups of students (not exclusively aimed at those with alerts).

System for evaluation, monitoring and strategies for the promotion of student engagement and contextual factors responds to this paradigm and the current needs at the country and regional level (IDB, 2021). The school engagement and contextual factors assessment instruments have been assessed on a small scale in other Latin American countries, and progress has been made in their validation and adjustments. Work was conducted in four Ibero-American countries: Spain, Peru, Colombia, and Uruguay (Lara et al., 2021). This allows us to point out future challenges to the internationalization of the system, posing the challenge and the opportunity to conduct consultancies and support the validation of the evaluation platform and the implementation of a system of evaluation, follow-up, and strategies for the promotion of school commitment in Spanish-speaking countries. A central challenge would be the technological debugging, consisting of an exhaustive review and adjustments to the technological architecture (servers and services) to adjust the web system to other countries and avoid collapses in the face of a massive use by schools geographically distributed in distinct parts of Chile and other Latin American countries.

However, based on the technological advantages of SIESE (resources provided and usability), it is necessary to ensure that certain essential conditions are in place for it to be used effectively in its implementation, as described in this article. The results and conclusions of this article point to the importance of continuing research on how information is organized, managed and used in educational centers (Soncin and Cannistrà, 2022) in the context of the progressive development of data systems for educational management (IDB, 2021).

Much has been published in Anglo-Saxon journals on psychosocial or psychoeducational interventions within schools and their impacts on a wide range of areas, including student behavior, school climate, school engagement and school retention, among others. These studies are still scarce in Latin America. One of the main challenges for future research in Chile and Latin America is the need to conduct studies with probability samples. Therefore, we suggest the need for quasi-experimental designs to study the effects of SIESE usability and implementation on educational trajectories and other outcomes. On the other hand, the time factor may have possible effects of achieving more decisive results considering that establishing trusting professional relationships between schools, families, and students (School Engagement Boards) take time and is key in these types of integrated interventions.

## Data availability statement

Publicly available datasets were analyzed in this study. This data can be found here: https://compromisoescolar.com/.

## Ethics statement

The studies involving human participants were reviewed and approved by the Comité de Ética Científico de la Universidad Autónoma de Chile. The patients/participants provided their written informed consent to participate in this study.

## Author contributions

MS, XT, and AR drafted the manuscript. MS and XT developed the study concept and the study design. MBS and AR performed the data collection. MS, XT, AR, and LL contributed to the interpretation of the data and provided important critical revision. All authors approved the final revision and also agreed to be accountable for all aspects of the work.

## Abbreviations

SIESE: System for Evaluation, Monitoring and Strategies for the Promotion of Student Engagement and Contextual Factors
SE: School Engagement
CF: Contextual Factors.
